# Aging germline stem cells in C. elegans

**DOI:** 10.1093/geroni/igaa057.2644

**Published:** 2020-12-16

**Authors:** E Jane Hubbard

**Affiliations:** Skirball/NYU, New York, New York, United States

## Abstract

Failure to maintain stem cells with age is associated with conditions such as tissue degeneration and increased susceptibility to tissue damage. We use the C. elegans germline stem cell system as a model to study stem cell aging. This system combines a well-established model for aging with an accessible stem cell system, providing a unique opportunity to understand how aging influences stem cell dynamics. The germline stem/progenitor pool in in C. elegans becomes depleted over time. At the cellular level, aging influences both the size of the stem cell pool and the proliferation rate of stem cells. The flux of differentiated cells also affects how aging impacts the pool. This depletion is partially alleviated in mutants with reduced insulin/IGF-like signaling via inhibition of the transcription factor DAF-16/FOXO. In this role, DAF-16 does not act in the germ line, and its anatomical requirements are different from its previously described roles in larval germline proliferation, dauer control, and lifespan regulation. We found that DAF-16/FOXO is required in certain somatic cells in the proximal part of the reproductive system to regulate the stem cell pool. We also find that the degree to which various age-defying perturbations affect lifespan does not correlate with their effect on germline stem cell maintenance. We are investigating additional aspects of aging germline stem cells using this system.

